# The Impact of Household and Community Indoor Residual Spray Coverage with Fludora Fusion in a High Malaria Transmission Setting in Northern Zambia

**DOI:** 10.4269/ajtmh.22-0440

**Published:** 2023-06-26

**Authors:** Ellen Ferriss, Mike Chaponda, Mbanga Muleba, Jean-Bertin Kabuya, James Sichivula Lupiya, Christina Riley, Anna Winters, Lawrence H. Moulton, Modest Mulenga, Douglas E. Norris, William J. Moss, for the Southern and Central Africa International Centers of Excellence for Malaria Research

**Affiliations:** ^1^ Department of International Health, Johns Hopkins Bloomberg School of Public Health, Baltimore, Maryland;; ^2^ Tropical Diseases Research Centre, Ndola, Zambia;; ^3^ Akros, Lusaka, Zambia;; ^4^ University of Montana, Missoula, Montana;; ^5^ Pfizer Canada, Quebec, Canada;; ^6^ Directorate of Research and Postgraduate Studies, Lusaka Apex Medical University, Lusaka, Zambia;; ^7^ W. Harry Feinstone Department of Molecular Microbiology and Immunology, Johns Hopkins Bloomberg School of Public Health, Baltimore, Maryland;; ^8^ Department of Epidemiology, Johns Hopkins Bloomberg School of Public Health, Baltimore, Maryland

## Abstract

Zambia’s National Malaria Elimination Program transitioned to Fludora Fusion in 2019 for annual indoor residual spraying (IRS) in Nchelenge District, an area with holoendemic malaria transmission. Previously, IRS was associated with reductions in parasite prevalence during the rainy season only, presumably because of insufficient residual insecticide longevity. This study assessed the impact of transitioning from Actellic 300CS to long-acting Fludora Fusion using active surveillance data from 2014 through 2021. A difference-in-differences analysis estimated changes in rainy season parasite prevalence associated with living in a sprayed house, comparing insecticides. The change in the 2020 to 2021 dry season parasite prevalence associated with living in a house sprayed with Fludora Fusion was also estimated. Indoor residual spraying with Fludora Fusion was not associated with decreased rainy season parasite prevalence compared with IRS with Actellic 300CS (ratio of prevalence ratios [PRs], 1.09; 95% CI, 0.89–1.33). Moreover, living in a house sprayed with either insecticide was not associated with decreased malaria risk (Actellic 300CS: PR, 0.97; 95% CI, 0.86–1.10; Fludora Fusion: rainy season PR, 1.06; 95% CI, 0.89–1.25; dry season PR, 1.21; 95% CI, 0.99–1.48). In contrast, each 10% increase in community IRS coverage was associated with a 4% to 5% reduction in parasite prevalence (rainy season: PR, 0.95; 95% CI, 0.92–0.97; dry season: PR, 0.96; 95% CI, 0.94–0.99), suggesting a community-level protective effect, and corroborating the importance of high-intervention coverage.

## INTRODUCTION

Two decades after the resumption of indoor residual spraying (IRS) in Zambia, malaria remains one of the nation’s leading public health challenges. In 2018, 30.4% of children younger than 5 years in the country’s highest burden province, Luapula, were parasitemic by microscopy.^1^ Despite an estimated household IRS coverage of 64.2% and a reported bed net use of 79.9%, under-five parasite prevalence has remained stable since 2012.^1^

Across Zambia, IRS has been deployed increasingly in high-burden rural areas, either alone or in conjunction with long-lasting insecticide-treated nets (LLINs) to maximize community impact.^2^^,^^3^ As of 2018, household IRS coverage had increased to 35% nationally.^1^ Although IRS in Zambia has generally been accompanied by reductions in malaria burden, campaign success has been variable. From 2006 through 2012, IRS coverage was associated with a 70% decrease in the odds of infection in children younger than 5 years across Zambia.^4^ More recently, IRS was associated with a 9% reduction in health facility–confirmed malaria case incidence in Eastern, Luapula, Muchinga, and Northern provinces, and a 25% reduction where operations were supported by the mSpray/Reveal mapping application.^5^

Despite the relative successes of IRS, spraying has proved vulnerable to insecticide resistance, necessitating the development of new products for the public health sector.^6^ In 2014, pirimiphos-methyl (Actellic 300CS, Syngenta AG, Basel, Switzerland) was introduced in Nchelenge District, Luapula Province—an area with holoendemic malaria and demonstrated insecticide resistance to both pyrethroids and carbamates.^7^ After the transition to Actellic 300CS for IRS, rainy season parasite prevalence was estimated to decrease 25% in the district’s sprayed areas.^8^ However, similar reductions were not observed during the dry season, suggesting insecticide residual activity was too short-lived to target the primary vector, *Anopheles funestus* sensu stricto, which peaks in abundance during the dry season.

Zambia’s National Malaria Elimination Program expanded available insecticides in 2018 with SumiShield (Sumitomo Chemical Co., Ltd., Tokyo, Japan), and in 2019 with Fludora Fusion (Bayer CropScience AG, St. Louis, MO). The latter, a novel formulation of the neonicotinoid clothianidin and the pyrethroid deltamethrin, was used in Nchelenge District beginning that year.^9^ Cone bioassays conducted locally in sprayed mud and concrete houses demonstrated 100% mortality of susceptible colonized *Anopheles gambiae* Kisumu strain mosquitoes within 6 days, up to 10 months after IRS.^10^ Fludora Fusion’s long residual duration was, thus, expected to improve annual IRS effectiveness in areas like Nchelenge District that experience holoendemic malaria transmission. This study is among the first to compare the impact of IRS with Fludora Fusion on parasite prevalence against an established insecticide under operational conditions.

## MATERIALS AND METHODS

### Study area.

Nchelenge District is located in northern Zambia in Luapula Province, adjacent to the southeastern border of the Democratic Republic of the Congo. The district abuts Lake Mweru and is situated amid marshland, lagoons, and islands, making it a well-suited habitat for multiple anopheline mosquito species and year-round malaria transmission.^11^ *Anopheles funestus*, the primary vector, peaks in abundance during the dry season, spanning May through October, and *An. gambiae*, which serves as a secondary vector, persists at low counts throughout the year.^11^ Both vectors are highly anthropophilic and endophagic.^12^ The human population is mobile, traveling regularly between densely populated, urban lakeside areas and inland farms.^13^

**Figure 1.**
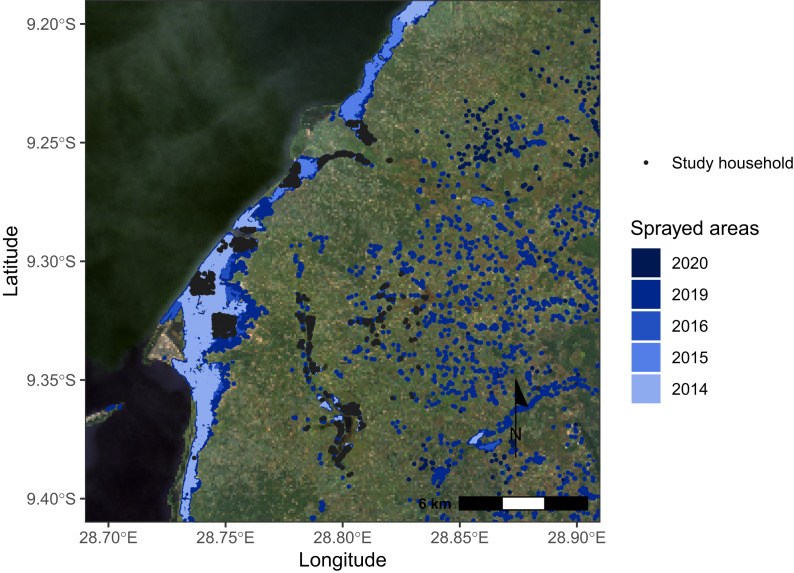
Study site depicting participating households and areas receiving indoor residual spraying from 2014 to 2021.

Malaria control in the district includes case management, chemoprevention during pregnancy, annual IRS, and LLIN distributions. Indoor residual spraying is conducted from late September through early December just prior to the rainy season; however, spraying is not timed to the seasonal peak abundance of *An. funestus*, potentially limiting its efficacy.^11^ From 2014 to 2016, IRS was targeted to household clusters with the greatest projected case counts—that is, those in health facility catchment areas with high incidences and high population densities.^14^ Thereafter, it was offered in all health facility catchment areas with year-over-year increases in the total area sprayed (Figure 1). Through 2018, LLINs were distributed every 3 years through mass campaigns aimed at achieving universal coverage (one net per two people) and, in addition, through antenatal and vaccination clinics on an ongoing basis.^15^ Since 2020, vector control has been implemented using a mosaic approach that provides households with either IRS or LLINs.^15^

### Data collection.

Community-based active surveillance was conducted monthly by the Southern and Central Africa International Center of Excellence for Malaria Research (ICEMR) from 2012 through 2021, as described elsewhere.^8^ Surveillance was paused from April through June 2020, January through March 2021, and July 2021, in accordance with internal and national safety protocols during the SARS-CoV-2 pandemic. This analysis used serial cross-sectional survey data—that is, data from households observed once and baseline data from households observed longitudinally, collected between November 2014 and August 2021. Data were processed and analyzed in R version 4.1.2 (R Core Team, Vienna, Austria).

In brief, study conduct was as follows. Satellite imagery was used to enumerate households that were selected for study inclusion via random sampling of 1-km × 1-km grid cells that were superimposed on the site map, with oversampling in low-population density cells. Questionnaires and malaria rapid diagnostic tests (RDTs) were administered to enrolled participants by study staff at each visit, and RDT-positive individuals were offered artemether/lumefantrine or, if in the first trimester of pregnancy, quinine and clindamycin. In addition, household mosquito collections were conducted both indoors and outdoors using CDC light traps (John W. Hock Co., Gainesville, FL). Study participants older than 16 years of age and caretakers of participants younger than 16 years provided written informed consent at the time of survey administration.

### Data.

#### Outcome of interest.

Parasite prevalence—that is, the proportion of RDTs that were positive—was estimated using participant results. Separate estimates were generated for the 2014 through 2020 rainy seasons, the 2015 through 2021 dry seasons, and the 2020 and 2021 dry seasons.

#### Exposures.

The exposures of interest were 1) spray period, 2) household spray status, 3) the interaction of the two (i.e., the differential effect of living in a sprayed house depending on the spray period [IRS insecticide]), and 4) annual IRS structure coverage across the site.

Spray period was classified according to the IRS insecticide used. November 2014 through September 2019 was coded as the Actellic 300CS period, and October 2019 through August 2021 as the Fludora Fusion period.

Household spray status was ascertained using survey data and the Reveal mapping application (previously mSpray) via Abt Associates. Self-reported household spray status (i.e., whether a household received IRS within the past 6 months) was obtained from the ICEMR survey for participants observed during the rainy season from 2014 through 2019. Self-reported household spray status was not considered during the dry season, as households sprayed in the most recent campaign typically received IRS more than 6 months prior to the survey and, thus, would not have reported receiving IRS. For the 2019 and 2020 spray years, household spray status was obtained from the U.S. President’s Malaria Initiative (PMI) program data collected by VectorLink and Akros through Reveal, enabling spray status capture during both the rainy and dry seasons.^16^ Spray status was identified by linking enumerated structures to ICEMR study households by visual comparison in QGIS version 3.22 LTR (QGIS Development Team, Gossau, Switzerland). The ICEMR study households absent from the Reveal data set were marked unsprayed, and those that could not be linked to a structure because of geocoding imprecision were assigned a spray status only if all possible matches—that is, equally close enumerated structures in the Reveal data set—were sprayed or unsprayed similarly. Self-reported spray status was used for households that could not be linked definitively.

Annual IRS structure coverage was defined as the percentage of ICEMR households reporting spray receipt—that is, reporting having received IRS—between the end of the annual IRS campaign up to 6 months after its commencement for the 2014 through 2018 spray years using an expanded data set that included the first observation from longitudinal households after each IRS campaign. Coverage for the 2019 and 2020 spray years was estimated as the percentage of ICEMR study structures marked as receiving IRS in the Reveal data set after the completion of annual IRS. Time since spraying was estimated as the number of weeks from the start of the annual campaign to the participant observation date less 10 days, which was an adjustment made to account for the lag in IRS impact, assuming a 10-day intrinsic incubation period.

#### Potential confounders.

Age, gender, LLIN use, the number of LLINs owned by the household, head of household educational attainment, household water source, floor type, roof type, the presence of open eaves, primary and secondary residence, and elevation were obtained from the ICEMR surveys. Primary residence was coded as inland (at least 3 km from Lake Mweru) or lakeside (within 3 km from Lake Mweru), with or without a secondary farming residence. Age was modeled using spline terms. Knots were selected by fitting serial Poisson regression models predicting RDT status and identifying those that minimized model quasi-likelihood under the independence model criterion (QIC).

Spatial and environmental data were obtained from publicly available sources. Shape files for Nchelenge District’s road network and Lake Mweru were obtained from the Humanitarian OpenStreetMap Team and were augmented through digitization of unrepresented features. Nchelenge District’s stream network was classified with the Strahler system in ArcGIS (Environmental Systems Research Institute, Redlands, CA) using the Arc Hydro tool with a 90-m resolution digital elevation model from the National Aeronautics and Space Administration’s (NASA’s) Shuttle Radar Topography Mission.^17^ Streams were designated as first through fifth order, with first-order streams having no feeders and each subsequent stream order being formed by the convergence of two similar lower order streams—that is, two first-order streams converged to form a second-order stream. Household distance to the nearest health facility, road, and stream, and to Lake Mweru were calculated in R statistical software using the sf package.^18^ One hundred-meter gridded population density data were obtained from GRID3.^19^ Local vegetation coverage—that is, the normalized difference vegetation index—was obtained at the household level within 10 days of participant observation from the EROS Moderate Resolution Imaging Spectroradiometer (version 6) 250-m resolution data, courtesy of the U.S. Geological Survey. Climatological data were obtained from the Climate Hazards Group Infrared Precipitation with Stations (CHIRPS) data set using the chirps R package and from the NASA Langley Research Center POWER Project funded through the NASA Earth Science Directorate Applied Science Program.^20^ Estimated daily rainfall from CHIRPS was used to calculate 7-day rolling rainfall averages that were then lagged 10 to 90 days from participant observation. Optimal lag times were identified by regressing RDT status on lagged average rainfall using serial Poisson regression models with robust variance estimation and household clustering with the geepack R package.^21^^–^^23^ Lag times that minimized model QIC were selected. When RDT status was best predicted by rainfall at more than one lag, multiple candidates were tested separately and concurrently in the final model. Daily 1/2° latitude and 5/8° longitude global grid temperature data from NASA were processed using the same methodology.

### Statistical analysis.

A difference-in-differences analysis was conducted to estimate the association between rainy season parasite prevalence and living in a house sprayed in the past 6 months, compared with an unsprayed house, allowing for differential impact depending on whether Fludora Fusion or Actellic 300CS was used. In addition, the association between parasite prevalence and community coverage with IRS was estimated. This analysis used the methodology described by Hast et al.,^8^ with the individual taken as the unit of analysis and the participant RDT result the observed outcome. The absence of data on IRS receipt for participants observed during the Actellic 300CS period dry seasons precluded a dry season difference-in-differences analysis. Instead, the association between parasite prevalence and living in a house sprayed with Fludora Fusion was estimated for participants observed during the 2020 and 2021 dry seasons. A separate model was fit to estimate the association between dry season parasite prevalence and community IRS coverage across all years. Poisson regression models with robust variance estimation were fit using generalized estimating equations with clustering at the household level. Models were adjusted for environmental, behavioral, and demographic characteristics identified previously as risk factors at the study site, in addition to characteristics associated with risk in preliminary data exploration.^8^^,^^24^

## RESULTS

A total of 4,499 participants from 1,410 households were surveyed from November 2014 through August 2021. Median participant age was 14 years old (quartile 1–quartile 3 [Q1–Q3] = 6–31), and 57% of the study population was female. Throughout the study, an estimated 56% of households received IRS during the most recent campaign, increasing from 51% during the years in which Actellic 300CS was used to 71% during the Fludora Fusion spray period. From 2014 through 2021, 64% of participants reported sleeping under a bed net. Most participants (82%) lived in structures with thatch roofs and open eaves. Participant characteristics according to spray period and household spray status are shown in Table 1 for the 2014 to 2020 rainy seasons, and in Table 2 for the Fludora Fusion spray period dry seasons (2020 and 2021).

**Table 1:** Participant characteristics during the 2014 to 2020 rainy seasons, according to the insecticide used during the most recent IRS campaign and household spray status

Characteristic	Actellic 300CS		Fludora Fusion	
	Sprayed (*n* = 838)	Unsprayed (*n* = 855)	Sprayed (*n* = 427)	Unsprayed (*n* = 172)
Age, years; median (Q1, Q3)	15.0 (6.0, 32.0)	14.0 (6.0, 30.0)	14.0 (6.0, 27.0)	13.5 (5.8, 28.0)
Female, *n* (%)	472 (56.3)	477 (55.8)	250 (58.5)	104 (60.5)
Weeks since IRS campaign, median (Q1, Q3)	12.9 (7.7, 18.1)	14.1 (5.1, 17.9)	13.3 (6.7, 22.4)	10.7 (8.8, 21.7)
Sleeps under LLIN, *n* (%)	606 (72.3)	514 (60.1)	243 (56.9)	80 (46.5)
No. of LLINs owned by family, median (Q1, Q3)	2.0 (1.0, 3.0)	2.0 (1.0, 2.0)	1.0 (0.0, 2.0)	1.0 (0.0, 2.0)
Head of household educational attainment, *n* (%)				
Primary	576 (68.7)	643 (75.2)	263 (61.6)	117 (68.0)
Secondary or higher	262 (31.3)	212 (24.8)	164 (38.4)	55 (32.0)
Water source, *n* (%)				
Improved, piped or borehole	478 (57.0)	402 (47.0)	262 (61.4)	78 (45.3)
Unimproved, open well or surface water	360 (43.0)	453 (53.0)	165 (38.6)	94 (54.7)
House roof material, *n* (%)				
Metal	146 (17.4)	117 (13.7)	125 (29.3)	31 (18.0)
Thatch	692 (82.6)	738 (86.3)	302 (70.7)	141 (82.0)
House eave type, *n* (%)				
Closed	63 (7.5)	53 (6.2)	67 (15.7)	9 (5.2)
Open	775 (92.5)	802 (93.8)	360 (84.3)	163 (94.8)
Residence, *n* (%)				
Primary, ≥ 3 km from Lake Mweru	196 (23.4)	374 (43.7)	77 (18.0)	107 (62.2)
Primary, < 3 km from Lake Mweru	180 (21.5)	180 (21.1)	63 (14.8)	10 (5.8)
Only, < 3 km from Lake Mweru	462 (55.1)	301 (35.2)	287 (67.2)	55 (32.0)
Population density, persons/100 m^2^; median (Q1, Q3)	27.4 (15.9, 40.6)	21.2 (11.5, 36.8)	18.8 (11.7, 33.3)	19.0 (11.0, 32.2)
No. of kilometers to health facility, median (Q1, Q3)	2.2 (0.7, 3.9)	3.6 (0.8, 6.4)	2.2 (0.8, 3.7)	4.4 (1.9, 7.0)
No. of kilometers to nearest road, median (Q1, Q3)	0.1 (0.0, 0.1)	0.1 (0.0, 0.1)	0.1 (0.0, 0.1)	0.1 (0.0, 0.2)
No. of kilometers to first-order stream, median (Q1, Q3)	0.4 (0.2, 0.6)	0.3 (0.2, 0.5)	0.4 (0.2, 0.6)	0.3 (0.2, 0.7)
No. of kilometers to second-order stream, median (Q1, Q3)	0.8 (0.4, 1.6)	0.6 (0.3, 1.4)	0.7 (0.4, 1.5)	0.6 (0.1, 1.2)
No. of kilometers to third-order stream, median (Q1, Q3)	2.6 (2.2, 3.4)	2.2 (0.8, 2.7)	2.7 (2.3, 3.8)	1.4 (0.4, 2.8)
No. of kilometers to fourth-order stream, median (Q1, Q3)	3.2 (0.6, 4.0)	3.3 (2.5, 4.1)	3.2 (2.6, 4.1)	3.3 (2.4, 4.0)
No. of kilometers to fifth-order stream, median (Q1, Q3)	7.9 (1.8, 9.2)	8.3 (5.7, 9.5)	6.1 (0.8, 9.2)	8.1 (5.3, 9.8)
Elevation, m; median (Q1, Q3)	944.5 (933.0, 955.0)	950.0 (938.0, 960.0)	944.9 (934.8, 957.3)	957.8 (938.0, 968.5)

IRS = indoor residual spraying; LLIN = long-lasting insecticide-treated net; Q1 = quartile 1; Q3 = quartile 3.

**Table 2:** Participant characteristics during the Fludora Fusion period dry seasons (2020 and 2021), according to household spray status

Characteristic	Sprayed (*n* = 333)	Unsprayed (*n* = 154)
Age, years; median (Q1, Q3)	13.0 (6.0, 25.0)	12.0 (5.0, 32.8)
Female, *n* (%)	194 (58.3)	83 (53.9)
Weeks since IRS campaign, median (Q1, Q3)	40.4 (31.9, 44.7)	40.9 (2.3, 47.1)
Sleeps under LLIN, *n* (%)	223 (67.0)	104 (67.5)
No. of LLINs owned by family, median (Q1, Q3)	2.0 (1.0, 2.0)	1.0 (1.0, 2.0)
Head of household educational attainment, *n* (%)		
Primary	220 (66.1)	99 (64.3)
Secondary or higher	113 (33.9)	55 (35.7)
Water source, *n* (%)		
Improved, piped or borehole	225 (67.6)	102 (66.2)
Unimproved, open well or surface water	108 (32.4)	52 (33.8)
House roof material, *n* (%)		
Metal	57 (17.1)	18 (11.7)
Thatch	276 (82.9)	136 (88.3)
House eave type, *n* (%)		
Closed	14 (4.2)	21 (13.6)
Open	319 (95.8)	133 (86.4)
Residence, *n* (%)		
Primary, ≥ 3 km from Lake Mweru	98 (29.4)	69 (44.8)
Primary, < 3 km from Lake Mweru	80 (24.0)	25 (16.2)
Only, < 3 km from Lake Mweru	155 (46.5)	60 (39.0)
Population density, persons/100 m^2^; median (Q1, Q3)	21.4 (13.0, 33.4)	23.9 (12.5, 33.4)
No. of kilometers to nearest health facility, median (Q1, Q3)	2.4 (0.8, 4.4)	3.3 (0.8, 6.7)
No. of kilometers to nearest road, median (Q1, Q3)	0.1 (0.0, 0.1)	0.1 (0.0, 0.1)
No. of kilometers to first-order stream, median (Q1, Q3)	0.4 (0.3, 0.7)	0.4 (0.1, 0.6)
No. of kilometers to second-order stream, median (Q1, Q3)	0.8 (0.2, 1.4)	0.9 (0.5, 1.4)
No. of kilometers to third-order stream, median (Q1, Q3)	2.6 (0.6, 3.6)	2.1 (0.4, 2.6)
No. of kilometers to fourth-order stream, median (Q1, Q3)	3.3 (2.3, 4.1)	3.2 (2.7, 3.8)
No. of kilometers to fifth-order stream, median (Q1, Q3)	7.6 (1.5, 9.7)	7.1 (5.4, 9.8)
Elevation, m; median (Q1, Q3)	947.1 (936.4, 953.7)	950.0 (940.1, 963.0)

IRS = indoor residual spraying; LLIN = long-lasting insecticide-treated net; Q1 = quartile 1; Q3 = quartile 3.

**Figure 2.**
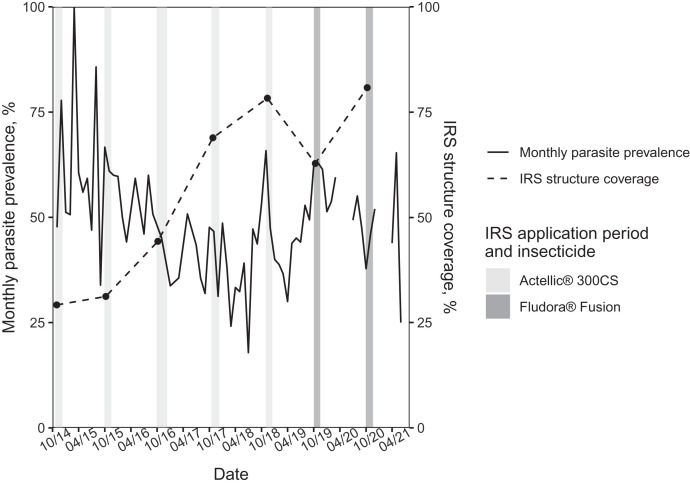
Monthly parasite prevalence and site IRS coverage from 2014 to 2021. IRS = indoor residual spraying.

Site IRS structure coverage increased from 29% during the 2014 spray campaign to 81% in 2020. Parasite prevalence declined from late 2014 through mid-2018 after the 2014 introduction of Actellic 300CS in IRS (Figure 2). Parasite prevalence averaged 45.8% (95% bootstrap confidence interval [BCI], 43.6–47.9) from November 2014 through September 2019 when Actellic 300CS was in use, and averaged 51.5% (95% BCI, 47.8–54.9) thereafter, during the Fludora Fusion spray period.

Household spray receipt in the past 6 months was not statistically significantly associated with decreased rainy season parasite prevalence during the Actellic 300CS spray period (prevalence ratio [PR], 0.97; 95% CI, 0.86–1.10) or the Fludora Fusion period (PR, 1.06; 95% CI, 0.89–1.25) (Table 3). Furthermore, the two insecticides were not associated differentially with risk (ratio of PRs, 1.09; 95% CI, 0.89–1.33). Unlike household spray status, increasing percent IRS coverage among ICEMR households across the site was associated with decreased rainy season parasite prevalence (PR, 0.95; 95% CI, 0.92–0.97), suggesting a community-level protective effect. No association with weeks since the annual IRS campaign and rainy season parasite prevalence was estimated (PR, 1.00; 95% CI, 0.99–1.02), indicating no measurable decline in residual activity across the rainy season. To assess possible bias imparted by differential misclassification of household spray status according to insecticide period, a sensitivity analysis was performed using self-reported spray status for both periods. Estimates for direct and indirect effects of IRS were comparable to those described (sensitivity analysis results not shown).

**Table 3:** Modeled rainy season parasite prevalence associated with living in a house that received IRS compared with living in a house that did not

Characteristic	Prevalence ratio	95% CI	*P* value
Fludora Fusion spray period compared with Actellic 300CS spray period	1.20	1.02–1.42	0.029
House unsprayed	Ref.	Ref.	Ref.
House sprayed in past 6 months with Actellic 300CS	0.97	0.86–1.10	0.640
House sprayed in past 6 months with Fludora Fusion compared with Actellic 300CS, ratio of prevalence ratios	1.09	0.89–1.33	0.409
Increase of 10% in site IRS coverage	0.95	0.92–0.97	< 0.001
Weeks since IRS campaign	1.00	0.99–1.02	0.599
Sleeps under LLIN	0.92	0.82–1.03	0.129
No. of LLINs owned by family	1.00	0.95–1.05	0.969
Age, years	1.06	1.04–1.08	< 0.001
Age, additional year beyond age 9	0.90	0.88–0.93	< 0.001
Age, additional year beyond age 28	1.02	1.00–1.03	0.033
Female	0.86	0.80–0.94	< 0.001
Secondary + education	0.86	0.76–0.97	0.014
Open eaves	1.16	0.89–1.50	0.269
Natural or unfinished floor	1.18	0.95–1.47	0.145
Thatch roof	1.05	0.82–1.34	0.700
Unimproved water source	0.95	0.85–1.06	0.392
Primary residence ≥ 3 km from Lake Mweru	1.00	–	–
Primary residence < 3 km from Lake Mweru	0.83	0.64–1.07	0.156
Only residence < 3 km from Lake Mweru	0.85	0.67–1.07	0.168
Population density, additional 10 persons/100 m^2^	0.96	0.93–0.99	0.019
Log kilometers to the nearest health facility	1.17	1.05–1.31	0.006
No. of kilometers to nearest road	1.10	0.71–1.68	0.675
No. of kilometers to first-order stream	0.77	0.64–0.92	0.003
No. of kilometers to second-order stream	1.01	0.92–1.11	0.803
No. of kilometers to third-order stream	1.01	0.92–1.10	0.888
No. of kilometers to fourth-order stream	0.96	0.91–1.01	0.114
No. of kilometers to fifth-order stream	0.95	0.91–0.98	0.003
No. of kilometers to fifth order stream beyond 9 km	1.12	1.01–1.25	0.040
Elevation, 10-m increase	0.98	0.96–1.01	0.130
76-Day lagged average daily rainfall, mm	0.97	0.94–1.00	0.095
38-Day lagged average minimum temperature, °C	1.03	0.98–1.10	0.252

IRS = indoor residual spraying; LLIN = long-lasting insecticide-treated net; Ref. = reference value.

Similarly, during the dry season, living in a house that received IRS with Fludora Fusion was not associated with a decreased risk compared with living in an unsprayed house (PR, 1.21; 95% CI, 0.99–1.48) (Table 4). On the contrary, participants living in sprayed houses were estimated to have increased risk of parasitemia, possibly reflecting greater acceptance of IRS among greater risk households. As during the rainy season, increasing IRS coverage among ICEMR households across the site was associated statistically significantly with decreased parasite prevalence (Fludora Fusion period: PR, 0.88; 95% CI, 0.79–0.99; all years: PR, 0.96; 95% CI, 0.94–0.99) (Table 5). No association between parasite prevalence and weeks since the IRS campaign was estimated.

**Table 4:** Modeled dry season parasite prevalence associated with living in a house that received IRS with Fludora Fusion compared with living in a house that did not

Characteristic	Prevalence ratio	95% CI	*P* value
House unsprayed	Ref.	Ref.	Ref.
House sprayed	1.21	0.99–1.48	0.062
Increase of 10% in site IRS coverage	0.88	0.79–0.99	0.034
Weeks since IRS campaign	1.00	0.99–1.00	0.688
Sleeps under LLIN	0.96	0.76–1.22	0.742
No. of LLINs owned by family	0.86	0.79–0.94	< 0.001
Age, years	1.06	1.02–1.10	0.004
Age, additional year beyond age 9	0.89	0.84–0.94	< 0.001
Age, additional year beyond age 28	1.05	1.01–1.09	0.027
Female	0.88	0.74–1.04	0.139
Secondary + education	0.92	0.76–1.11	0.405
Open eaves	2.02	0.73–5.60	0.176
Thatch roof	0.95	0.70–1.29	0.765
Unimproved water source	1.06	0.81–1.39	0.663
Population density, 10 persons/100 m^2^	0.99	0.94–1.04	0.627
Log kilometers to the nearest health facility	0.95	0.80–1.13	0.568
No. of kilometers to first-order stream	1.01	0.75–1.37	0.941
No. of kilometers to second-order stream	1.11	0.97–1.26	0.125
No. of kilometers to third-order stream	0.77	0.63–0.94	0.010
No. of kilometers to fourth-order stream	1.01	0.93–1.10	0.751
No. of kilometers to fifth-order stream	0.91	0.84–0.98	0.012
No. of kilometers to fifth-order stream beyond 9 km	1.08	0.90–1.31	0.398
41-Day lagged average daily rainfall, mm	1.10	1.05–1.15	< 0.001

IRS = indoor residual spraying; LLIN = long-lasting insecticide-treated net; Ref. = reference value.

**Table 5:** Modeled dry season parasite prevalence associated with community IRS coverage with Actellic 300CS or Fludora Fusion

Characteristic	Prevalence ratio	95% CI interval	*P* value
Increase of 10% in site IRS coverage	0.96	0.94–0.99	0.008
Weeks since IRS campaign	1.00	0.99–1.00	0.249
Sleeps under LLIN	0.86	0.78–0.95	0.004
No. of LLINs owned by family	0.96	0.91–1.01	0.100
Age, years	1.06	1.05–1.08	< 0.001
Age, additional year beyond age 9	0.89	0.87–0.91	< 0.001
Age, additional year beyond age 28	1.04	1.02–1.06	< 0.001
Female	0.90	0.83–0.97	0.005
Secondary + education	0.98	0.87–1.09	0.655
Open eaves	1.28	0.90–1.81	0.167
Natural or unfinished floor	1.02	0.85–1.22	0.833
Thatch roof	1.22	0.95–1.56	0.115
Unimproved water source	0.97	0.88–1.08	0.621
Primary residence ≥ 3 km from Lake Mweru	1.00	–	–
Primary residence < 3 km from Lake Mweru	0.81	0.62–1.06	0.119
Only residence < 3 km from Lake Mweru	0.88	0.68–1.15	0.358
Population density, 10 persons/100 m^2^	1.00	0.97–1.03	0.830
Log kilometers to the nearest health facility	1.05	0.96–1.16	0.256
No. of kilometers to nearest road	0.78	0.53–1.15	0.218
No. of kilometers to first-order stream	0.81	0.69–0.96	0.015
No. of kilometers to second-order stream	1.09	0.99–1.20	0.067
No. of kilometers to third-order stream	0.97	0.89–1.06	0.491
No. of kilometers to fourth-order stream	0.98	0.94–1.03	0.524
No. of kilometers to fifth-order stream	0.94	0.91–0.97	< 0.001
No. of kilometers to fifth-order stream beyond 9 km	1.18	1.08–1.29	< 0.001
87-Day lagged maximum temperature, °C	0.97	0.94–1.01	0.128
24-Day lagged average daily rainfall, mm	1.04	1.01–1.07	0.012

IRS = indoor residual spraying; LLIN = long-lasting insecticide-treated net.

Potential confounders associated with parasitemia included age, gender, head of household educational attainment, LLIN use, the number of LLINs in the household, population density, proximity to a health facility and various order streams, and average daily rainfall. Malaria risk increased in children from infancy to 9 years (rainy season: PR, 1.06; 95% CI, 1.04–1.08; dry season, all years: PR, 1.06; 95% CI, 1.05–1.08) and decreased thereafter. Female gender was associated with a lower parasite prevalence (rainy season: PR, 0.86; 95% CI, 0.80–0.94; dry season, all years: PR, 0.90; 95% CI, 0.83–0.97), as was secondary school completion by the head of household (rainy season: PR, 0.86; 95% CI, 0.76–0.97; dry season, all years: PR, 0.98; 95% CI, 0.87–1.09). Self-reported LLIN use was associated with decreased parasite prevalence during the dry season only (rainy season: PR, 0.92; 95% CI, 0.82–1.03; dry season, all years: PR, 0.86; 95% CI, 0.78–0.95), indicating a greater LLIN impact several months after IRS—that is, when residual insecticide may no longer be active on walls and when indoor vector abundance peaks. No association was estimated during the Fludora Fusion period dry seasons (PR, 0.96; 95% CI, 0.76–1.22), presumably reflecting deteriorated LLIN quality after the final mass distribution campaign in 2017 and 2018. The number of LLINs owned by the household was associated with decreased parasite prevalence during the Fludora Fusion dry seasons only (PR, 0.86; 95% CI, 0.79–0.94), possibly suggesting a relationship between the number of nets in the household and more recent net acquisition. Housing construction characteristics, including roof type, floor type, presence of open eaves, household water source, and location of residency, were not statistically significantly associated with parasitemia after adjusting for other confounders. However, built and natural environmental characteristics—namely, urbanicity and hydrology measures—were associated with risk. During the rainy season, a greater population density was associated with lower parasitemia (PR, 0.96; 95% CI, 0.93–0.99), likely reflecting an indirect protective effect of neighboring structure IRS coverage. Increased distance to the nearest health facility was associated with increased parasitemia during the rainy season (PR, 1.17; 95% CI, 1.05–1.31), whereas household distance to the nearest first- and fifth-order streams was associated negatively with risk (rainy season: PR, 0.77; 95% CI, 0.64–0.92; PR, 0.95; 95% CI, 0.91–0.98, respectively; dry season, all years: PR, 0.81; 95% CI, 0.69–0.96; PR, 0.94; 95% CI, 0.91–0.97, respectively). During the dry season, lagged average daily rainfall was associated with a greater parasite prevalence (Fludora Fusion period: PR, 1.10; 95% CI, 1.05–1.15; all years: PR, 1.04; 95% CI, 1.01–1.07).

## DISCUSSION

The transition from Actellic 300CS to Fludora Fusion for annual IRS in Nchelenge District was expected to reduce the risk of parasitemia in sprayed households by extending the length of time that residual insecticide activity deterred vectors from entering structures. However, the findings of this study did not indicate a benefit of living in a house sprayed with either insecticide in this holoendemic malaria setting. Experimental hut trials in eastern Africa have previously demonstrated high initial deterrence of *An. funestus* s.l. and *An. gambiae* s.l. from entering structures sprayed with pirimiphos-methyl (Actellic) and neonicotinoids, suggesting reduced indoor exposure to potentially malarial mosquitoes.^25^ However, the duration of residual activity, as estimated by vector mortality, has varied widely, suggesting declining insecticide deterrence.^26^ Pirimiphos-methyl residual activity on mud walls induced at least 80% mortality for 2 to more than 9 months after IRS across East and West Africa, and 4 to 5 months in Zambia in routine program monitoring.^26^ In experimental hut trials, pirimiphos-methyl was estimated to achieve greater than 80% mortality for less than 3 months and only 50% mortality by 7 to 8 months after spraying.^25^ Similarly, Fludora Fusion demonstrated declining residual activity in experimental hut trials in Cove, Benin, despite showing greater than 80% vector mortality for up to 10 months in cone bioassays in Nchelenge District and Cove.^10^^,^^27^ Vector mortality declined from more than 90% 1 month after IRS application to less than 20% after 11 months.^27^ Other studies have shown shorter residual duration of Fludora Fusion in cone bioassays. In Dangbo, Benin, and Gujarat, India, residual activity induced at least 80% mortality for only 6 months (72-hour mortality) and 7 months (120-hour mortality), respectively, which is far shorter than the duration needed in a setting with year-round transmission.^28^^,^^29^ Unpublished vector data from the ICEMR study site indicate reduced indoor vector abundance in structures sprayed with either insecticide during the rainy season but not the dry season, suggesting waning residual activity. While vector data point to declining insecticide deterrence, parasite prevalence was not associated with weeks since the most recent IRS campaign, potentially reflecting the insensitivity of parasite prevalence to declines in indoor vector abundance in this setting.

Baseline differences in risk between households that received IRS and those that did not may have confounded the association of parasite prevalence and household spray status. Areas with greater projected malaria case counts were targeted for spraying during the first 3 years Actellic 300CS was used.^14^ In addition, households with a greater risk may have been more likely to accept IRS than lower risk households within sprayed areas. Thus, the direct protective effect of IRS may have been underestimated as a result of confounding by risk factors that were not included in this analysis. Importantly, the dry season risk associated with living in a house sprayed with Fludora Fusion may be an overall reduction beyond what would have been observed had the house been sprayed with a shorter-acting insecticide or not sprayed at all.

In contrast to household IRS receipt, community spray coverage was associated with a statistically significant decrease in risk during the rainy and dry seasons. Previous research at the site has estimated comparable reductions in rainy season parasite prevalence among individuals from sprayed and unsprayed households within IRS-targeted areas (33% and 26%, respectively), suggesting a predominantly community-level protective effect of IRS.^8^ Contemporaneous research in the region estimated a 25% decrease in facility-reported incidence rates in catchments receiving IRS supported by mSpray—that is, those achieving greater coverage.^5^ Furthermore, a 70% decrease in odds of infection among children younger than 5 years of age was estimated for higher coverage areas across Zambia.^4^ The protective effect of high community coverage has also been noted in Equatorial Guinea and Malawi, where living in an area with at least 80% coverage and 50% to 80% coverage (although not higher) was associated with decreased malaria risk, whereas living in a sprayed house was associated with little to no reduction in the odds of parasitemia.^30^ Finally, a randomized controlled trial in Northeast Tanzania estimated a 90% reduction in the entomological inoculation rate (EIR) for individuals from both sprayed and unsprayed households in villages that received IRS.^31^ These findings emphasize the importance of high IRS coverage for achieving community-level protection.

Despite year-over-year gains in IRS coverage, which exceeded 70% during the 2018 and 2020 campaigns, a sustained decline in parasite prevalence was not observed. Increased estimated risk during the Fludora Fusion period may have resulted from secular trends in vector abundance, vector control, and health-care access. Parasite prevalence increased from 2018, possibly as a result of increasing indoor vector counts (unpublished data) and declining LLIN use beginning that year. Mass LLIN distribution, previously conducted every 3 years, ended with the 2017 and 2018 campaign, likely resulting in decreased net quality by the time of the transition to Fludora Fusion, as suggested by PMI research estimating a 1.5-year net life span and the absence of an estimated reduction in parasite prevalence associated with LLIN use during the Fludora Fusion period.^32^ Furthermore, IRS structure coverage fell to less than 60% in 2019, which was likely low enough to limit a mass effect. Last, malaria commodity stockouts arising from underinvestment in 2019 and supply chain disruptions in 2020 during the COVID-19 pandemic may have exacerbated increasing transmission.^32^

This study highlights the limitations of annual IRS in Nchelenge District. The area’s stable parasite prevalence, despite the switch to a new long-acting insecticide and improved IRS coverage, is likely attributable to the interplay of insufficient insecticide duration, misalignment of spray campaigns with peak vector abundance, high year-round vector counts, and possible exophily and exophagy facilitated by human behaviors, such as outdoor resting in farming areas. Indoor residual spraying has, historically, been conducted between the end of the dry season and the early rainy season—several months preceding the increase in *An. funestus*. Thus, campaign timing has likely limited both household and community benefit during the dry season. Shifting IRS to the end of the rainy season could target peak vector abundance more effectively. However, biannual spraying is likely necessary to sustain reductions in transmission if long-acting insecticides do not provide year-round protection. The stability of parasite prevalence should also be considered in light of the force of infection. Previous research has estimated a local EIR exceeding 80 infectious bites per person per year, well above the rate necessary to sustain a parasite prevalence of 50%.^12^^,^^33^ For IRS to lower parasite prevalence effectively, it would likely need to reduce the annual EIR to less than 10 infectious bites per person per year.^33^ Indeed, research and unpublished vector data from the site have demonstrated a lower abundance of *An. funestus* and total anophelines in houses sprayed with either insecticide, suggesting a direct, albeit insufficient, impact.^34^ Last, although IRS has been effective in high-transmission areas, including southern Mozambique and northeastern Tanzania, a high impact may be unattainable in the presence of exophily and exophagy (M. Gebhardt, personal communication).^31^^,^^35^ Outdoor resting of blooded anophelines has been posited as a likely impediment to earlier IRS campaigns in the Sudan Savanna and has been documented at rates consistently exceeding 80% and 60% in eastern African hut trials of Actellic and pyrethroid IRS, respectively.^25^^,^^36^

## CONCLUSION

Fludora Fusion use in the 2019 and 2020 IRS campaigns was not associated with decreased rainy season parasite prevalence among individuals residing in sprayed compared with unsprayed houses relative to previous years, when Actellic 300CS was used. Moreover, living in a house sprayed with either insecticide was not associated strongly with risk of parasitemia during the rainy or subsequent dry season. This study may have failed to capture differences in insecticide performance adequately due to the absence of a pronounced, direct protective effect. In contrast to household spray status, community IRS coverage was associated positively with a lower risk of parasitemia. Despite increasing coverage, parasite prevalence remained relatively stable throughout the study, indicating additional strategies for malaria control are necessary. Shifting IRS campaign operations to the end of the rainy season to target the primary vector more effectively may improve IRS impact, although, ideally, two rounds of annual IRS should be considered. In addition, universal LLIN coverage and more frequent deployment are indicated. Although the WHO does not recommend IRS-LLIN codeployment, particularly in place of delivering either intervention at high coverage, these findings suggest codeployment may provide an additive benefit.^7^^,^^37^ Effective nonpyrethroid IRS and pyrethroid-only LLIN codeployment has been demonstrated previously in Zambia, Sudan, Tanzania, and Mozambique, supporting cointervention as a possible strategy.^38^^–^^42^ However, as Nchelenge District transitions from pyrethroid to pyrethroid-piperonyl butoxide (PBO) LLINs, restored vector susceptibility to LLIN insecticides may render IRS unnecessary.^41^ In keeping with WHO recommendations, improving coverage of either intervention should be prioritized.^37^ Last, future malaria control activities should include operational research to identify which interventions are most effective and which, if any, can be discontinued safely.
